# Response of *Fragaria vesca* to projected change in temperature, water availability and concentration of CO_2_ in the atmosphere

**DOI:** 10.1038/s41598-023-37901-8

**Published:** 2023-07-01

**Authors:** Iris Sammarco, Zuzana Münzbergová, Vít Latzel

**Affiliations:** 1 Institute of Botany, Czech Academy of Sciences, Průhonice, Czechia; 2 Department of Botany, Faculty of Science, Charles University, Prague, Czechia

**Keywords:** Gene expression, Environmental impact, Ecology, Physiology, Plant sciences, Ecology

## Abstract

The high rate of climate change may soon expose plants to conditions beyond their adaptation limits. Clonal plants might be particularly affected due to limited genotypic diversity of their populations, potentially decreasing their adaptability. We therefore tested the ability of a widely distributed predominantly clonally reproducing herb (*Fragaria vesca*) to cope with periods of drought and flooding in climatic conditions predicted to occur at the end of the twenty-first century, i.e. on average 4 °C warmer and with twice the concentration of CO_2_ in the air (800 ppm) than the current state. We found that *F. vesca* can phenotypically adjust to future climatic conditions, although its drought resistance may be reduced. Increased temperature and CO_2_ levels in the air had a far greater effect on growth, phenology, reproduction, and gene expression than the temperature increase itself, and promoted resistance of *F. vesca* to repeated flooding periods. Higher temperature promoted clonal over sexual reproduction, and increased temperature and CO_2_ concentration in the air triggered change in expression of genes controlling the level of self-pollination. We conclude that *F. vesca* can acclimatise to predicted climate change, but the increased ratio of clonal to sexual reproduction and the alteration of genes involved in the self-(in)compatibility system may be associated with reduced genotypic diversity of its populations, which may negatively impact its ability to genetically adapt to novel climate in the long-term.

## Introduction

Keeping up with the ongoing climate change is undoubtedly a challenge for many organisms, not least plants. Their sessile lifestyle often impedes them from escaping to more favourable conditions within a generation^1,2^. The rate of climate change is also very likely faster than plants’ potential to genetically adapt to these changes^3^. Clonal plants, i.e. plants that reproduce by means of clonal reproduction in addition to sexual reproduction, might be particularly vulnerable under such circumstances because they usually form populations with limited standing genotypic variation, which can remarkably slow rates of their adaptation to the rapidly changing environment^4^. Additionally, clonal plants tend to favour habitats with higher moisture levels and lower mean annual temperatures compared to species that reproduce exclusively through sexual reproduction^5–7^, which make them particularly vulnerable to the increasing temperature and aridity. In this context, phenotypic plasticity seems to be a potentially crucial mechanism facilitating successful resistance of clonal plants to changing environment from the short- as well as long-term perspective^8^, thus preventing them from extinction. Although clonal plants are a dominant component of many ecosystems^9^, they have been relatively understudied in terms of their responses to predicted climate change. Therefore, research in this area should be of high priority.

Generally accepted models of the IPCC predicted that global mean temperature would increase up to 4 °C compared to the 1980–1999 period and CO_2_ concentration should rise up to 500–1260 ppm by the end of the twenty-first century^10^. These changes will be accompanied by increased fluctuations of water availability characterised by increasing frequency of drought periods^11,12^ along with higher evapotranspiration driven by a warmer environment^13^ and heavy precipitation events^14^. Recent studies have revealed that the growth and phenology of plants can change in response to increasing temperature (e.g.^15–18^). However, in most cases it is not known whether the observed plastic changes allow maintaining fitness^19^. Doubts also exist whether plasticity can enable adaptation to the future temperature that can be outside the range of physiological resistance of the plants (e.g.^18–20^). Elevated CO_2_ usually promotes biomass production and resistance to drought, nevertheless it can also have an antagonistic effect when interacting with other environmental factors^21,22^. As plants will be challenged by a multifactorial change, it is crucial to study their response in this perspective to refine our predictions and models.

Considering the multifaceted challenges plants will face due to climate change, it is imperative to expand our understanding of their responses beyond just phenology, reproduction, and growth, and delve into the underlying physiological and molecular mechanisms that drive the response to climate change. Recent advances in genomic methods enabled exploring plant responses to a changing climate at the molecular level. Transcriptome analysis, for instance, is a very powerful tool for identifying genes involved in responses to environmental stress and changing environmental conditions^23–26^. Transcript profiling of plants exposed to predicted environmental change, such as increased temperature or CO_2_ concentration, can thus provide us with a list of genes that are underpinning plant adaptation to climate change. Such a knowledge increases our understanding of the molecular basis of plant potential to cope with changing climate and allows to identify target genes for improving resistance of crop plants to future climatic conditions (e.g.^27^). While we have seen an increase in the number of studies dealing with physiology, growth or gene expression in response to climate change, we are not aware of any study that combined research at all the three levels. Nonetheless, to accurately predict how plants will function in the future, it is essential to integrate their responses to a multifaceted environmental change, including factors such as temperature, CO_2_ concentration, and water availability at the level on the growth, physiology, and gene expression.

Here we tested the response of a non-model clonal herb *Fragaria vesca,* the woodland strawberry, at the level of phenology, growth, physiology and gene expression to drought and flooding under temperature and CO_2_ conditions predicted to be prevalent at the end of the twenty-first century. More specifically, we cultivated plants in three distinct environments: one with ambient temperature and CO_2_ levels (400 ppm), another with a mean temperature increase of 4 °C and ambient CO_2_ level, and a third with both a mean temperature increase of 4 °C and elevated CO_2_ levels of 800 ppm (e[CO_2_]). This setup allowed us to investigate both the impact of warming on its own and in combination with e[CO_2_] on plant responses under varying water availability (i.e., drought or repeated flooding), compared to current environmental conditions. However, we could not evaluate the effect of CO_2_ independently—that is, without an increase in temperature—because we did not include a treatment with elevated CO_2_ levels only. We hypothesized that (1) Warming in combination with e[CO_2_] will trigger stronger positive or negative change in plant performance and gene expression than warming alone, and (2) the negative effects of drought or flooding on plants will be mitigated by e[CO_2_].

The benefits of utilising *F. vesca* as a model in our study are apparent in its ability to combine both reproductive strategies, thereby facilitating comparisons of ecological shifts in preference towards either strategy in response to environmental changes. This may enhance our understanding of the evolutionary benefits of each strategy.

## Material and methods

*Fragaria vesca* L., Rosaceae is a perennial clonal herb occurring in variety of disturbed habitats across the northern hemisphere. It can be found in regions with warm summers in northern Spain and Italy as well as in relatively cold regions of Scandinavia. It is able to reproduce both clonally by producing stolons and sexually through seeds (preferentially outcrossed but self-pollination is also possible), although its realized sexual reproduction in natural conditions is much rarer than clonal reproduction, and mainly occurs in disturbed areas where seedlings can establish a new population without being outcompeted^28,29^. As the study is part of a larger project involving a broad array of molecular analyses, we worked with a single nearly isogenic line Fb2:39–47, *F. vesca* cv. Reine des Vallées. The line was created by introgressing the runnering wildtype allele from *F. bucharica* into near-isogenic non-clonal line *F. vesca* ‘Reine des Vallées’ (‘RdV’) *tfl1*^30^. As a result of the introgression, the line produces abundant fruits but has also vigorous clonal growth.

In January 2020, seeds produced by controlled self-fertilisation of parental plants were stratified at 4 °C for 2 weeks before being transferred to sterilised sand for germination either under ambient or elevated CO_2_ (see below), all seeds germinated in ambient temperature. After 5 weeks, the seedlings were prepared for the main experiment. In April 2020, a total of 216 plants were individually grown in 30 × 40 × 8 cm trays filled with a commercial soil substrate specifically designed for strawberry cultivation (AGRO Substrát pro jahody). We planted a single seedling of *F. vesca* into the centre of each tray and subjected them to different temperature conditions and water availability. The use of plants in the present study complies with international, national and/or institutional guidelines.

### Temperature and CO_*2*_ manipulation

**Figure 1:**
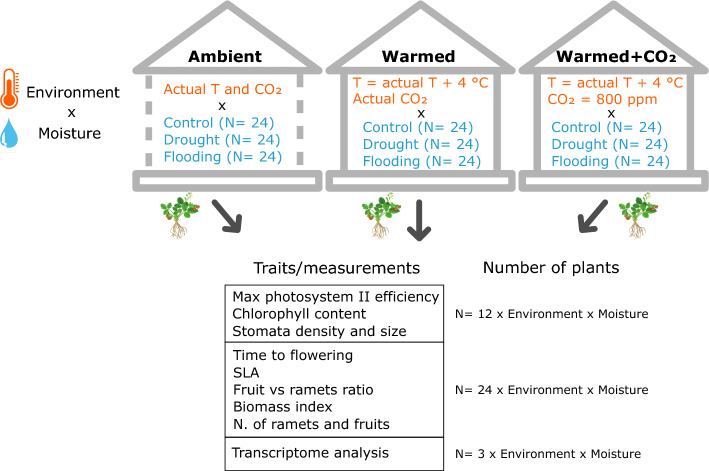
Experimental design used in this study. We grew the plants in in three different environments (in orange) (Ambient, Warmed and Warmed + CO_2_) and manipulated soil moisture in each environment (in blue) (control, drought and flooding). The Ambient environment was achieved thanks to an open greenhouse (greenhouse without side walls). N: number of plants.

The study was carried out from April to July 2020 at the Institute of Botany of the Czech Academy of Sciences in Průhonice, in two independent cubicles of a greenhouse and in a neighbouring greenhouse without side walls, further referred to as an open greenhouse. The two independent cubicles, each covering a 30 m^2^ area, were air conditioned. Custom-developed equipment and software were used to control the temperatures (either heating or cooling the air) to be on average 4 °C higher than the ambient temperature in the open greenhouse (Ambient temperature). The maximal temporal difference allowed between the greenhouse and ambient temperatures was set to 8 °C. In the first cubicle, we manipulated the concentration of CO_2_ in the atmosphere to be 800 ppm via automatic enrichment with commercial CO_2_ provided by Linde Gas company whereas we did not manipulate CO_2_ concentration in the second cubicle. Due to the logistical constraints of an open greenhouse, we were unable to manipulate CO_2_ levels in the ambient temperature treatment. We therefore created three different environments: actual temperature and actual CO_2_ concentration (further referred to as *Ambient* environment, N = 72), increased average temperature for 4° (further referred to as *Warmed* environment, N = 72) and increased average temperature for 4° as well as increased level of CO_2_ in atmosphere (further referred to as *Warmed* + *CO*_*2*_ environment, N = 72) (Fig. 1).

### Soil moisture

In addition to the manipulation of temperature and CO_2_ level, we also manipulated soil moisture (further referred to as moisture) (Fig. 1). One third of plants in all environments experienced *drought periods* (further referred to as drought treatment, 24 plants per environment). Those plants were watered with 200 ml of water only when they exhibited significant water stress, indicated by wilting leaves. On average, plants from the drought treatment in Ambient and Warmed environment experienced 9 drought cycles (i.e., watered 9 times after leaves wilted), while plants in the Warmed + CO_2_ treatment underwent 11 drought cycles. Another third of the plants had their trays submerged in water (with the water level aligned with the tray's soil surface) for 10 days, repeated three times during the study (referred to as the flooding treatment, 24 plants per environment). Each flooding event was followed by a 25-day recovery period. The remaining plants were watered when needed to keep the soil sufficiently wet and served as controls (24 plants per environment).

### Measurements

The temperature of the greenhouse and ambient cultivation was recorded on a 15-min basis by Thermologger TMS^31^. During the experiment, the mean temperature in the Ambient environment was 16.17 °C, the extreme minimal/maximal temperature was − 1.38/35.5 °C. Mean temperature in the Warmed environment was 20.14 °C, the extreme minimal/maximal temperature was 5.16/41.5 °C. Mean temperature in the Warmed + CO_2_ environment was 20.21 °C, the extreme minimal/maximal temperature was 4.75/41.2 °C.

The main study started in April and terminated on 20th July 2020, 20 days after we terminated flooding and/or drought treatments, i.e. all plants experienced control water treatment. We recorded time to first flowering of all plants. In July 2020, we estimated above ground biomass (further referred to as *biomass index*) of all maternal ramets (original seedlings) by multiplying the number of their leaves with the length of their longest leaf. The biomass index served as a reliable proxy for the actual biomass of the studied plants, enabling non-destructive estimation of above-ground biomass among all plants, as the experiment is part of a long-term study. In a separate study involving 453 individuals of the same line, we proved that the biomass index is highly correlated with the total dry biomass of *F. vesca* plants, including offspring ramets produced within the year of establishment (R^2^ = 0.747, F = 1332.234, P < 0.0001). We also assessed specific leaf area (SLA) of one fully developed leaf of every maternal ramet (expressed as a leaf area (cm^2^) divided by its dry mass (g)). We assessed the fresh leaf area by a Licor scanner and dry mass after drying at 80 °C for 24 h. At the same time, we recorded the number of ramets and number of fruits produced by each plant. We calculated the ratio between fruit and ramet number to test for the potential shift in investment to sexual and clonal reproduction in different environments and water availability levels.

Moreover, a week before termination of the study, we randomly selected half of the plants from each temperature and moisture treatment combination (N = 12 per temperature and moisture treatment) for which we measured maximum photosystem II (PSII) efficiency (Fv/Fm), chlorophyll content, stomatal density and stomatal length. These measurements were taken on plants that were not currently experiencing stress, as stress treatments were suspended a week prior to the measurements. In the case of the drought treatment, plants were water-saturated, and for the flooding treatment, plants were not flooded. This approach ensured that any observed differences could be attributed to the long-term response to different treatments, rather than to stress actively affecting the plants at the time of measurement.

We measured maximum photosystem II efficiency (Fv/Fm) using fluorimeter FluorPen FP-100 MAX/USB (Photon System Instruments, Czechia) on 1 h dark-adapted plants^32^. We measured three fully developed leaves of every maternal ramet (i.e. the original planted seedling), and the final value was average of the three measurements. The ratio Fv/Fm is considered to be a good indicator of overall photosynthetic capacity of plants^33,34^ with values around 0.8 representing healthy leaves/plants.

The leaf chlorophyll content (Cab μg/cm^2^) was determined using a CCM-300 chlorophyll content meter^35^. The average obtained from three measured leaves of each plant was considered.

We used epidermal impressions of the bottom site of leaves made with clear nail polish to assess stomatal density and stomatal length. Clear nail polish was applied to the underside of a leaf, allowed to dry completely, and then carefully peeled off using transparent tape. The resulting film was then mounted on a microscope slide for further investigation. The stomatal density was assessed by averaging the number of stomata in three not overlapping areas (each 250 × 250 µm). Each counting area was located in the middle part of a leaflet. We also measured the length of 3 randomly selected stomata in each of the counting area. We averaged the stomatal density and stomatal length for every measured leaf (one leaf per plant).

### Transcriptome analysis

We collected leaf samples from 3 randomly selected plants from each treatment combination (27 samples together) 3 days after we measured photosynthesis. Samples were frozen in liquid nitrogen and kept in − 80 °C until RNA extraction. We extracted mRNA using the Nucleospin RNA Plus kit (Macherey Nagel) according to the manufacturer’s instructions with minor modifications. In order to improve RNA quality and yield from *F. vesca*, a known recalcitrant species, we used an increased amount of lysis buffer (500 µl) together with 100 µl of EDTA (0.5 M, pH = 8) and PVPP (polivinilpolipirrolidone). A cDNA library for RNA-sequencing was constructed and sequenced PE150 using an Illumina NovaSeq 6000 platform by Novogene Co., Ltd, Cambridge. We trimmed adaptors with the command-line tool cutadapt (v1.16) and assessed sequencing quality with MultiQC (v1.10.1)^36^. We aligned the reads to the *F. vesca* genome (v4.0.a2) using STAR (Spliced Transcripts Alignment to a Reference) (v2.7.1a)^37^, and assembled them into transcripts using StringTie (v2.1.5)^38^. We identified differentially expressed genes (DEGs) between different environments and treatments with the DESeq2 package for R (v1.30.1)^39^. For identification of DEGs, we considered as thresholds for statistical significance an adjusted P value < 0.05 (default method for adjusting P values, Benjamini-Hochberg) and an absolute value of fold change (FC) ≥ 1.5. We then performed Gene Ontology (GO) enrichment analysis of DEGs with the clusterProfiler package for R (v3.18.1)^40^, which performs a hypergeometric test comparing the set of significantly enriched genes against all the background genes. We used an FDR adjusted P value < 0.05 as threshold for statistical significance.

### Statistical analysis

The effect of environment, moisture and their interaction on single plant traits was tested using general linear models for most traits as they were fitting Gaussian distribution after possible transformations (log transformation of biomass index, time to flowering and number of ramets, sqrt transformation of fruit to ramet ratio; the residuals of the models were not deviating from Gaussian distribution after inspection of result diagnostic plots). The exception was number of fruits tested using generalised linear model and following Poisson distribution. We used the “dispersiontest” function implemented in AER package in R version 3.6.2 to test for overdispersion in the number of fruits and identified that the values are not over dispersed and assuming Poisson distribution for testing this variable is reasonable. We used Tukey's HSD to estimate the pairwise differences among treatments.

To investigate the relationship between gene expression and plant traits, we used redundancy analysis (RDA) performed in vegan package version 2.6-2^41^ in R. The plant trait data (i.e. traits related to physiology, growth and fitness) were transformed as mentioned above and standardized for 0 mean and unit variance (to transfer all the traits into relative, i.e. comparable, units) and were used as dependent variables. We used the transformed trait values to ensure that these trait value match those entering the GLM analyses used above. We used the expression level of all the differentially expressed genes as predictors. We used forward stepwise selection procedure to select the set of genes best predicting variation in the traits (selecting the gene with highest explanatory power and adding it in case of significant, continue selecting a next trait until the selected traits are significant). Significance of the effects was tested using permutation tests with 499 permutations of the data. The graphs accompanying the multivariate analyses have been drawn using CANOCO 5^42^.

## Results

Our analysis revealed that the environment treatment had a significant impact on all measured plant traits but stomata density and stomata size (Table 1). Moisture levels had a significant impact on all plant traits except for time to flowering. However, the effect of environment and moisture were in most cases not independent as their effects were interactive on all measured traits, except for Fv/Fm, chlorophyll index, stomata density, and time to flowering.

**Table 1:** The effect of environment, moisture, and their interaction on individual plant traits.

Trait	Environment		Moisture		Environment x moisture	
	F	p	F	p	F	p
Fv/Fm	5.53	**0.005**	12.62	** < 0.001**	0.67	0.612
Chlorophyll index	5.80	**0.004**	132.99	** < 0.001**	1.52	0.203
Stomata density	2.57	0.081	30.36	** < 0.001**	1.81	0.134
Stomata size	1.70	0.187	5.20	**0.007**	3.01	**0.022**
Time to flowering	50.73	** < 0.001**	0.91	0.405	0.29	0.882
Biomass index	63.27	** < 0.001**	171.98	** < 0.001**	2.84	**0.025**
SLA	140.35	** < 0.001**	137.65	** < 0.001**	6.56	** < 0.001**
Fruits/ramets	22.67	** < 0.001**	40.02	** < 0.001**	2.44	**0.048**
No. of ramets	66.67	** < 0.001**	94.52	** < 0.001**	12.75	** < 0.001**
No. of fruits	752.00	** < 0.001**	3409.70	** < 0.001**	163.80	** < 0.001**

Significant values (P < 0.05) are highlighted in bold.

### Growth performance

**Figure 2:**
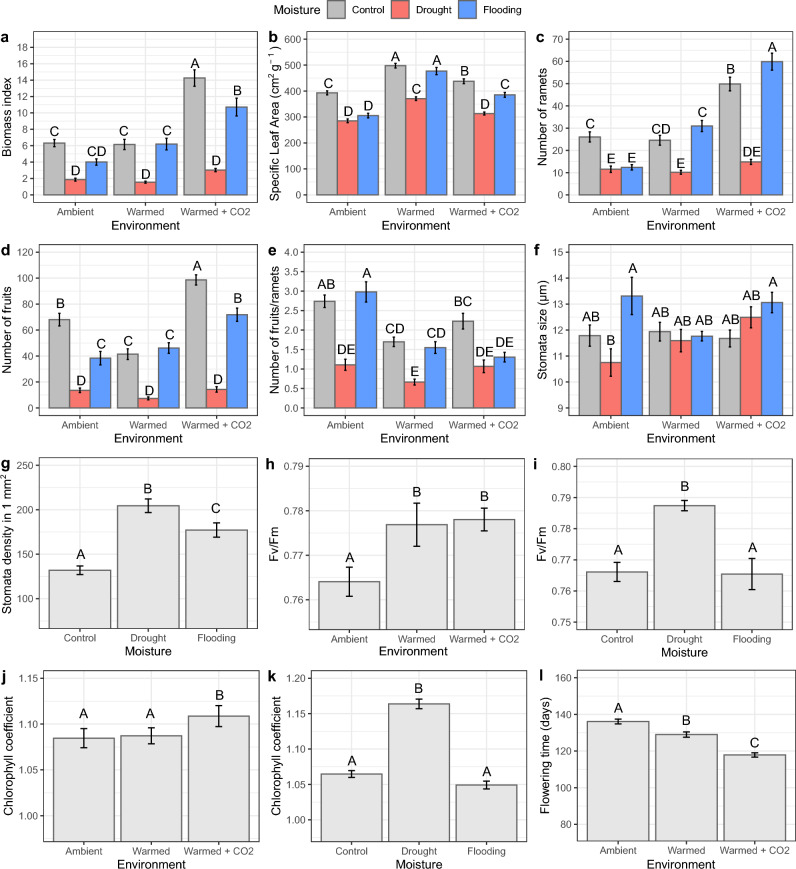
Plant traits measurements of *F. vesca* grown under different environment and/or moisture treatments. Only significant effects (according to Table 1) are shown. (**a**) Biomass index of maternal ramets (number of leaves × length of the longest leaf), (**b**) specific leaf area of maternal ramets, (**c**) number of offspring ramets and (**d**) fruits, (**e**) the ratio of produced fruits vs ramets, (**f**) stomata size, (**g**) stomata density, (**h**,**i**) maximal photosystem II efficiency (Fv/Fm), (**j**,**k**) chlorophyll content, and (**l**) flowering time. Maternal ramet refers to the original ramet developed from the seedling. Mean values are presented with error bars representing the ± 1 standard error (SE). Columns sharing the same letter are not significantly different from each other at P < 0.05 (Tukey HSD test).

Biomass index in Warmed + CO_2_ environment significantly differed from the Ambient and Warmed environments (Fig. 2a). *Fragaria vesca* increased biomass production in control and flooding moisture under Warmed environment and e[CO_2_], but did not change growth in drought condition in comparison to other treatments. Biomass index was also higher in flooded plants in Warmed environment in comparison to flooded plants in Ambient environment (Fig. 2a). SLA increased both in Warmed and Warmed + CO_2_ environments, with the increase being higher in Warmed environment (Fig. 2b). SLA was lower in drought and flooding moisture in comparison to control moisture in Ambient environment but SLA of flooded plants in Warmed and Warmed + CO_2_ environments was comparable to the control plants (Fig. 2b).

### Reproduction

Plants in Warmed + CO_2_ environment produced more offspring ramets and fruits than plants in other environments (Fig. 2c,d). Warmed + CO_2_ environment resulted in a considerable increase of the number of ramets and fruits in control and flooding treatment, but had no effect on ramets or fruits in drought treatment in comparison to Ambient environment (Fig. 2c,d). On the other hand, fruit number decreased in Warmed environment in control water regime in comparison to Ambient environment (Fig. 2d). The ratio between fruits and ramets decreased more in Warmed environment (Fig. 2e). Similar trend, although not significant, was observed in Warmed + CO_2_ environment in comparison to Ambient environment (Fig. 2e). This response outlined that clonality had tendency to be promoted over sexual reproduction in elevated temperature in our study system.

### Stomata and photosynthesis

Environment had different effects on stomata size in different moisture treatments. Stomata tended to be larger in Ambient drought treatment in comparison to plants from Ambient control treatment (Fig. 2f). Stomata density was instead affected only by moisture. Plants from drought had higher stomata density compared to plants from control treatment (Fig. 2g).

Maximal photosystem II efficiency (Fv/Fm) was higher both in Warmed only and Warmed + CO_2_ environment than in Ambient environment (Fig. 2h). Furthermore, Fv/Fm was highest in plants from drought treatment (Fig. 2i). Warmed environment did not alter the chlorophyll content in leaves but Warmed + CO_2_ environment significantly increased the chlorophyll content (Fig. 2j). Moreover, chlorophyll content was higher in plants from drought treatment (Fig. 2k).

### Flowering time

Environment significantly influenced flowering time. Compared to plants in Ambient environment, those in Warmed environment flowered approximately 7 days earlier, while those in Warmed + CO_2_ environment flowered about 18 days earlier (Fig. 2l).

### Differentially expressed genes (DEGs)

**Figure 3:**
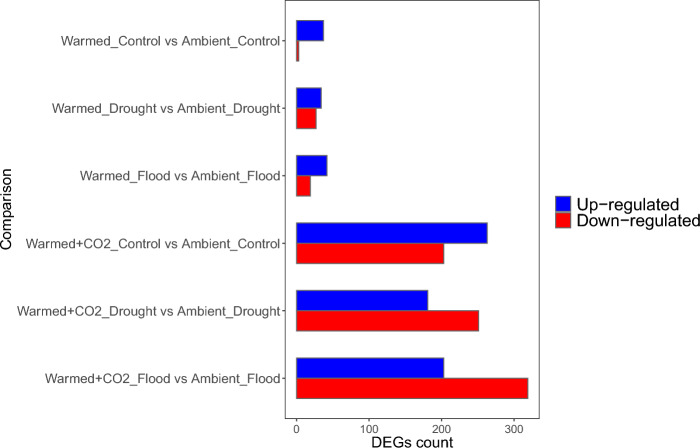
Number of up- and down-regulated differentially expressed genes (DEGs) identified in the comparisons between each environment and moisture to its respective Ambient environment.

To investigate the transcriptomic response of *F. vesca* to different environment and moisture treatments, we compared the gene expression profile of each environment and moisture treatment to its respective Ambient environment. We found most of the differentially expressed genes (DEGs) in the comparisons between the Warmed + CO_2_ and Ambient environment and their respective moisture (Fig. 3). In particular, in the Warmed + CO_2_ treatment comparisons, we found 463 DEGs (263 up-regulated, 203 down-regulated) in Ambient control treatment, 432 DEGs (181 up-regulated, 251 down-regulated) in Ambient and drought treatment, and 522 DEGs (230 up-regulated, 319 down-regulated) in ambient and flood treatment. In the Warmed environment comparisons, we found 40 DEGs (37 up-regulated, 3 down-regulated) in Ambient control treatment, 61 DEGs (34 up-regulated, 27 down-regulated) in ambient and drought treatment, and 61 DEGs (42 up-regulated, 19 down-regulated) in ambient and flood treatment.

**Figure 4:**
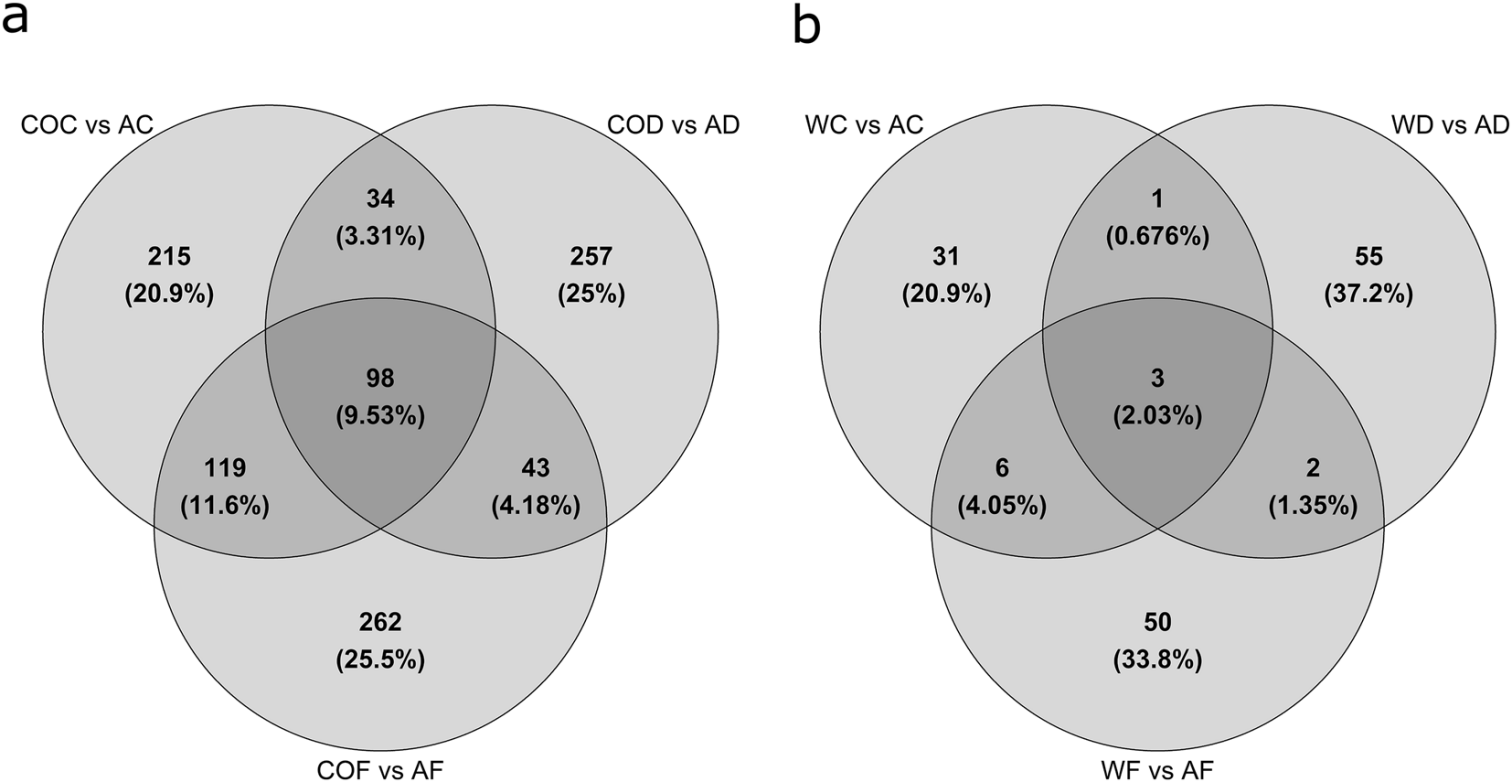
Number and percentage of differentially expressed genes (DEGs) overlapping among the comparisons between Warmed + CO_2_ and their respective Ambient environment in (**a**) and Warmed and their respective Ambient environment in (**b**). (**a**) COC vs AC: Warmed + CO_2__Control vs Ambient_Control, COD vs AD: Warmed + CO_2__Drought vs Ambient_Drought, COF vs AF: Warmed + CO_2__Flood vs Ambient_Flood. (**b**) WC vs AC: Warmed_Control vs Ambient_Control, WD vs AD: Warmed_Drought vs Ambient_Drought, WF vs AF: Warmed_Flood vs Ambient_Flood.

Among the DEGs found in the Warmed + CO_2_ comparisons, less than 10% were common to all conditions, while greater percentages of DEGs were unique to the control, drought and flood moisture, respectively (Fig. 4a). In the Warmed environment comparisons, we found only 2.03% of DEGs common to all conditions, and greater percentages of DEGs unique to the control, drought and flood moisture, respectively (Fig. 4b). When comparing the DEGs found between the Warmed + CO_2_ and Warmed environment comparisons, we found only 1% of DEGs common between control and drought, and drought and flood, and 12%, 72.4% and 13.5% unique to the control moisture, drought and flood, respectively.

**Figure 5:**
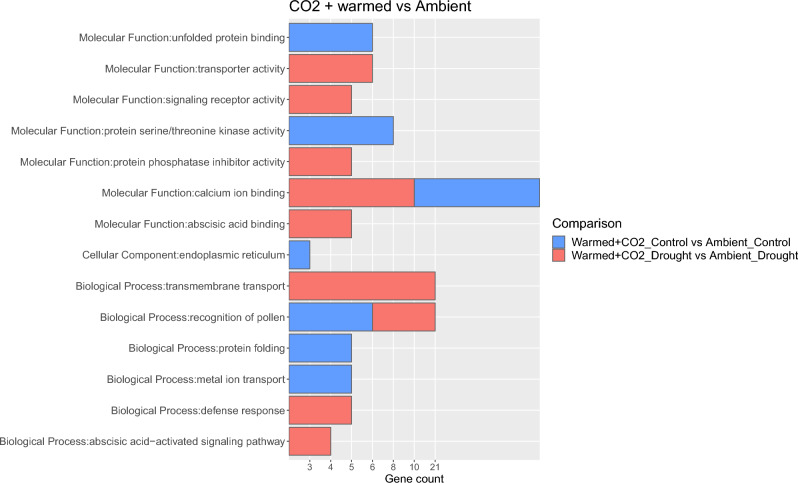
Gene Ontology (GO) enrichment analysis for the differentially expressed genes (DEGs) found in the comparisons between Warmed + CO_2_ and their respective Ambient environment. Gene count represents the number of genes assigned to each GO category. Only GO terms with an adjusted P value < 0.05 are shown.

GO enrichment analysis of the DEGs identified between each environment and its respective Ambient environment revealed no significantly up-regulated enriched terms for the DEGs identified in the comparisons between the Warmed environment and Ambient environment and their respective moisture levels, and for the Warmed + CO_2_ environment plus flood and Ambient environment plus flood. We found, however, several enriched GO terms for the DEGs found between Warmed + CO_2_ plus control moisture and Ambient environment plus control moisture, and Warmed + CO_2_ plus drought and Ambient environment plus drought (Fig. 5). In particular, we found two terms common to both comparisons, calcium ion binding (molecular function, GO:0005509; Ambient control: 10 DEGs, adjusted P = 0.019; Ambient drought: 10 DEGs, adjusted P = 0.020) and recognition of pollen (biological process, GO:0048544; Ambient control: 6 DEGs, adjusted P = 0.014; Ambient drought: 5 DEGs, adjusted P = 0.048). For the DEGs identified in the control environment comparison, we found also protein folding (biological process, GO:0006457; 5 DEGs, adjusted P = 0.045), metal ion transport (biological process, GO:0030001; 5 DEGs, adjusted P = 0.045), endoplasmic reticulum (cellular component, GO:0005783; 3 DEGs, adjusted P = 0.045), unfolded protein binding (molecular function, GO:0051082; 6 DEGs, adjusted P = 0.018) and protein serine/threonine kinase activity (molecular function, GO:0004674; 8 DEGs, adjusted P = 0.019). Finally, for the DEGs identified in the drought environment comparison, we found transmembrane transport (biological process, GO:0055085; 21 DEGs, adjusted P = 0.004), abscisic acid-activated signalling pathway (biological process, GO:0009738; 4 DEGs, adjusted P = 0.004), defence response (biological process, GO:0006952; 5 DEGs, adjusted P = 0.030), abscisic acid binding (molecular function, GO:0010427; 5 DEGs, adjusted P < 0.001), signalling receptor activity (molecular function, GO:0038023; 5 DEGs, adjusted P < 0.001), protein phosphatase inhibitor activity (molecular function, GO:0004864; 5 DEGs, adjusted P < 0.001) and transporter activity (molecular function, GO:0005215; 6 DEGs, adjusted P = 0.041).

### Gene expression correlation with trait groups

**Figure 6:**
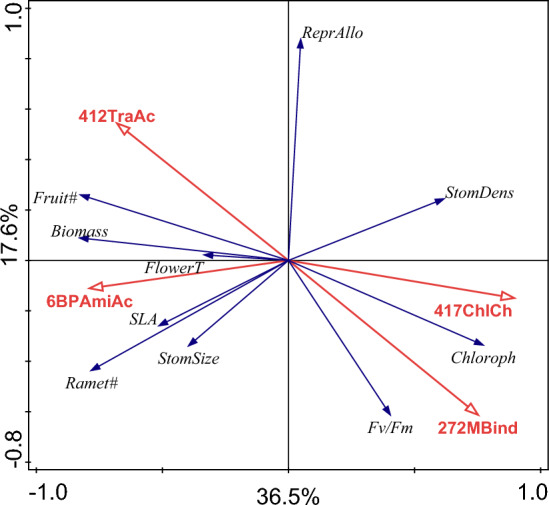
Relationship between the subset of Gene Ontology (GO) terms enriched in differentially expressed genes (DEGs) selected using forward step-wise selection procedure and the measured traits as determined using RDA analysis. Measured traits are represented in blue: ReprAllo: number of fruits/ramets; StomDens: stomata density; Chloroph: chlorophyll; Fv/Fm: maximal photosystem II efficiency; StomSize: stomata size; Ramet#: number of ramets; SLA: specific leaf area; FlowerT: flowering time; Biomass: biomass index; Fruit#: number of fruits. GO categories are represented in red: 417ChlCh: voltage-gated chloride channel activity; 272MBind: heme binding; 6BPAmiAc: aromatic amino acid family metabolic process; 412TraAc: ubiquitin-protein transferase activity.

We identified 4 different GO terms by step-wise selection performed using the DEGs most closely related to the studied traits. Our selection method involved identifying the GO term with the highest explanatory power, and adding it to the model only if it had a significant effect. We then repeated the procedure until the next GO term with the highest explanatory power was no longer significant. To investigate which gene expression change was correlated with trait response, we plotted GO terms enriched in DEGs to trait response to the environment and moisture treatments (Fig. 6). Such an approach enabled us to identify several gene categories that were positively correlated with individual trait categories. We found that the molecular function ubiquitin-protein transferase activity (412TraAc) was associated with number of fruits, biomass index and time to flowering. The ubiquitin–proteasome system regulates protein stability, mediates plant responses to environmental signals and plays a key role in many plant developmental stages, such as seed dormancy and germination and flowering time^43^. The biological process aromatic amino acid family metabolic process (6BPAmiAc) was mainly associated to time to flowering, biomass index, SLA and number of ramets. This GO term includes genes involved in pathways of the aromatic amino acids, which are linked to the synthesis of several secondary metabolites such as phytohormones^44^. Phytohormones are known to regulate many processes such as flowering time and biomass production^41,45^. We found the molecular function voltage-gated chloride channel activity (417ChlCh) to be associated with chlorophyll content and stomata density. This GO term includes genes regulating voltage-gated chloride channel activity, which control for example stomatal movement, nutrient transport and metal tolerance. The best documented examples are the chloride channels of guard cells, which control opening and closing of stomata. Finally, we found the molecular function heme binding (272MBind) to be associated with chlorophyll coefficient and Fv/Fm. Heme derives from the same biosynthetic pathway as chlorophyll, and it plays a crucial role in photosynthesis^42^.

## Discussion

Climate change represents a real threat to the existence of plants. Populations of clonal plants, in comparison to exclusively sexually reproducing plants, may be at a higher risk, as their ability to genetically adapt to new conditions is often diminished. Phenotypic plasticity has been therefore suggested as a key mechanism for clonal plants to cope with changing climatic conditions. In this study, we employed *Fragaria vesca*, a well-known clonal species with the ability to reproduce both clonally and sexually, as our model organism. This unique characteristic allowed for valuable insights into the roles of both reproductive strategies in response to anticipated future climate scenarios, encompassing increased temperature, elevated atmospheric CO_2_ concentration, and varying water availability.

### Higher temperature together with elevated CO_*2*_ had stronger effects on plants than warming alone

Overall, it was the Warmed + CO_2_ environment that triggered the strongest plastic response mainly in biomass index and number of ramets and fruits (Fig. 2). Positive effect of warming on flowering time (i.e. time until plants flower) belongs among the most reported responses of plants to climate warming^45–48^. On the other hand, the effect of e[CO_2_] on flowering time can range from positive to negative^49^. Previous studies have shown that when e[CO_2_] and increased temperature act simultaneously, their effects can be additive^50^ or interactive^51^, where e[CO_2_] can virtually eliminate the positive effect of warming on flowering time. In our study, plants flowered 7 days earlier under Warmed environment and about 18 days earlier in Warmed + CO_2_ treatment. This indicates that future changes in phenology of *F. vesca* will be influenced not only by the increasing temperature but even more by the combination of temperature increase and e[CO_2_]. Advanced time of flowering due to higher temperature and e[CO_2_] can considerably alter ecosystem functioning as it can modify interactions with pollinators^52^. We found flowering time to be linked to the differentially expressed genes (DEGs) belonging to the Gene Ontology (GO) terms ubiquitin-protein transferase activity and aromatic amino acid family metabolic process (Fig. 6). Protein ubiquitination is known to play a key role in many developmental stages, including flowering time (e.g.^53^). Analogously, aromatic amino acids are linked to the synthesis of phytohormones, which are important for the regulation of flowering time^41^.

Plants also grew bigger and had higher reproductive output in Warmed + CO_2_ environment than in Warmed environment, which is in accordance with other studies^54^. Biomass production and fitness related traits (i.e. number of fruits and number of ramets for ubiquitin-protein transferase activity and aromatic amino acid family metabolic process, respectively) were associated to the same GO terms mentioned above. Interestingly, protein ubiquitination has been shown to regulate leaf senescence under high CO_2_ conditions^55^ and phytohormones play a role in plant response to high CO_2_^56^. These genes were thus highly likely involved in molecular machinery enabling *F. vesca* to cope with increased temperature and to utilise increased level of atmospheric CO_2_.

Based on the relatively low number of DEGs between Warmed and Ambient environment in comparison to the Warmed + CO_2_ and Ambient environment, we deduce that the strongest effect on gene expression can be ascribed to the combined effect of a warmer temperature and e[CO_2_]. Our results are in strong contrast with a study on maize that found most of the DEGs between Warmed only and Ambient environment whereas very low DEGs between Warmed + CO_2_ and Ambient environment^57^. Such discrepancy can be likely ascribed to the fact that *F. vesca* is a C3 species whereas *Zea mays* is C4 species, i.e. species with different physiological and molecular pathways during the dark reaction of photosynthesis. That is, regarding the photosynthesis and consequent carbon uptake, e[CO_2_] should have stronger effect on C3 than on C4 species because C4 plants have already higher concentration of CO_2_ inside their specialised bundle sheath cells than the ambient CO_2_ concentration in Rubisco of C3 plants^58,59^. Hence, it is probable that the difference in photosynthesis mechanism between *F. vesca* and *Z. mays* can have direct or indirect (e.g. through the carbohydrate metabolism pathway) effects on the gene expression under e[CO_2_].

The GO functional enrichment analysis assigned most genes to the molecular function and biological process categories. E[CO_2_] affected mostly genes related to stress response (calcium ion binding, defence response, transmembrane transport) and reproduction (recognition of pollen). The identification of DEGs involved in pollen recognition and defence response belongs perhaps among the most interesting findings of our study. Genes involved in pollen recognition trigger a downstream molecular cascade resulting ultimately in rejection of pollen, which is a powerful molecular mechanism preventing self-pollination^60,61^. Genes primarily involved in defence response can also be activated by the pollen growing in the female tissue as it causes damage or stress to a plant^62^. In our study, under high e[CO_2_] plants generally downregulated genes involved in regulation of plant self-compatibility or defence, such as those encoding for S-locus lectin protein kinase family proteins and MLP-like protein 423 (see Table S1). This finding suggests that plants of *F. vesca* increased the level of self-pollinating in response to the e[CO_2_] through the alteration of gene expression. However, such interpretation has to be taken cautiously as it is based on the transcriptome analysis from leaves, not directly from flowers. Considering that we also found increased investment to clonal than sexual reproduction in Warmed environment, it can be speculated that the alteration of self-compatibility together with the increased clonal reproduction can be another important factor that can alter *F. vesca* populations to adapt to future environmental conditions, particularly under increased level of CO_2_. Levin^63^ already proposed that plants could promote self-pollination and clonality over cross-pollination strategy as a response to climate warming due to inadequate pollinator services expected under increased temperatures in the future. This is in line with Doležal et al.^64^ who documented an increased clonal reproduction over sexual reproduction of natural populations of *Rumex alpinus* in response to elevated annual mean temperatures. The shift towards selfing and/or clonal reproduction can significantly reduce genetic diversities of clonal plant populations in the future, which in turn can increase the probability of genetic bottleneck during extreme events^64–67^ and thus also their ability to genetically adapt to a changing environment. Therefore, although clonal populations could acclimatise to novel climate by phenotypic plasticity resulting even in increased overall fitness as found in our study, climate change can pose existential risk from the longer time perspective due to constrained genetic adaptability of clonal plant populations to further climatic conditions.

### Elevated atmospheric CO_*2*_ concentration does not increase drought tolerance but can facilitate response to temporal waterlogging

Elevated CO_2_ has been linked to the enhanced resistance of plants to drought due to higher photosynthetic efficiency that should contribute to improved water use efficiency^68^, an effect we did not observe in our study. Based on the biomass production, it can be even concluded that elevated CO_2_ very likely lowered water use efficiency in our study. Plants of the drought treatment from the Warmed + CO_2_ environment received on average 20% more water volume than plants from the drought treatment of Warmed environment to produce comparable biomass, number of fruits and ramets (see Fig. 2). It corresponds with studies demonstrating that strong drought can wipe out all beneficial effects of e[CO_2_] on plant water use efficiency (e.g.^69^).

Soil waterlogging is a stressful situation for most terrestrial plants. In general, flooding usually leads to hypoxia and ultimately to anoxia that strongly alter metabolism, growth, and survival of roots^70,71^. Interestingly, we found increased tolerance (increased growth and reproductive output) to temporal flooding in Warmed + CO_2_ environment compared to no tolerance to it in Ambient environment. Usually, the positive effect of e[CO_2_] on plant growth and reproduction can be reset by the wet environment^72^, which is apparently not the case of our study. Considering that the extent of the negative effect of flooding on plants depends on the timing and duration of the event^73^, it is evident that the period of the flooding was long and severe enough to be stressful for plants from the Ambient environment (see Fig. 2). The positive role of e[CO_2_] on plants ability to cope with temporal flooding can be explained by the overall higher water uptake of plants in e[CO_2_] environment when compared to warmed only environment due to their higher growth and with that related higher evapotranspiration. Clonal plants, in general, are much more successful in aquatic and waterlogged habitats than non-clonal species^74^. The enhanced capability of clonal plants to tolerate repeated waterlogging under e[CO_2_] can potentially even promote their success over non-clonal plants in wet habitats in the not so distant future. Since dry habitats are already dominated by non-clonal species^74^, it would be interesting to compare the effect of e[CO_2_] on water use efficiency between clonal and non-clonal plants.

It is worth mentioning that we present data from a relatively short time exposure of plants to changed environmental conditions. Plants can be able to adapt to e[CO_2_] if provided sufficient time, if their populations are exposed to e[CO_2_] for multiple generations, which could allow for selection of favourable genotypes^26,75^. On the other hand, populations of clonal plants can lack high standing genetic variation and thus their ability to genetically adapt to rapidly increasing e[CO_2_] can be limited. Moreover, photosynthetic measurements in our study revealed that plants were already acclimatized to e[CO_2_]. Therefore, we consider our study relevant providing valuable information on the ability of the species to cope with future environmental conditions.

## Conclusion

In conclusion, our study provides valuable and unique insights into the response of a model clonal plant species, *Fragaria vesca*, under a combination of climate change factors, including warming, elevated CO_2_ concentration, and varying water availability. Our findings suggest that *F. vesca* can successfully adjust its phenotype to predicted future climate conditions, however increased clonality of the species under Warmed environment suggest reduced genetic adaptability to the changed climate in the longer time perspective. We demonstrated that elevated CO_2_ and increased temperature has a stronger effect on *F. vesca*'s growth, reproductive output, and gene expression than warming alone and revealed a previously undocumented positive effect of e[CO_2_] on the plant's ability to cope with temporal but repeated waterlogging. Future studies focusing on climatic change should thus account for the effect of elevated CO_2_ to avoid potentially inaccurate conclusions and thus also misleading predictions.

Moreover, thanks to the detailed knowledge of the change in gene expression in response to climate change, we found that *F. vesca* alters the level of self/outcrossing ratio in response to elevated CO_2_ concentration through gene expression, suggesting a potential adaptive strategy for future environmental conditions.

Our findings contribute to a growing body of literature on plant responses to climate change by focusing on the complex interactions of multiple climate change factors and their effects on a clonal plant species. This study increases our understanding of the potential impacts of climate change on plant communities, ecosystems, and their functioning, such as altered interactions with pollinators and the balance between clonal and non-clonal reproduction in different habitats. Further research on the long-term effects of climate change factors on clonal and non-clonal plants, as well as the potential genetic adaptations and consequences for ecosystem functioning, will be crucial for understanding and mitigating the impacts of climate change on plant communities and the environment.

Last but not least, we showed that plant response to climate change is a very complex process and the exploration of a wide variety of plant responses is key to obtain general insights into plants future. In fact, only individual measurements, such as physiological traits in our study, would not provide us with sufficient data to be able to make more general predictions.

## Supplementary Information

Supplementary Table S1.

## Data Availability

The data were deposited in the European Nucleotide Archive (ENA, http://www.ebi.ac.uk/ena/) under the reference number PRJEB51906.
