# Supplementary material for: Response of Fragaria vesca to projected change in temperature, water availability and concentration of CO2 in the atmosphere
Source: Sci Rep. 2023 Jul 1;13:10678. doi: 10.1038/s41598-023-37901-8 (PMC10314927; doi:10.1038/s41598-023-37901-8)
Supplement: Supplementary file 1 — Supplementary Table S1. [file 41598_2023_37901_MOESM1_ESM.docx]

**Supplementary material**

**Table S1.** Differentially expressed genes (DEGs) found in the comparisons between warmed+CO2 and their respective ambient temperature treatment, enriched in the biological processes Gene Ontology (GO) terms recognition of pollen **(a)** and defence response **(b)**. COC vs AC: Warmed+CO2_Control vs Ambient_Control, COD vs AD: Warmed+CO2_Drought vs Ambient_Drought, COF vs AF: Warmed+CO2_Flood vs Ambient_Flood. A negative log_2_(FC) signifies a lower expression in Warmed+CO2 respect to its respective ambient temperature treatment. Values in bold are significant at both adjusted P < 0.05 and fold change (FC) ≥ 1.5 (corresponding to log_2_(FC) ≥ 0.58).

| **(a)** **Recognition of pollen** | |  |  |  |  |  |  |
| --- | --- | --- | --- | --- | --- | --- | --- |
|  |  | COC vs AC | | COD vs AD | | COF vs AF | |
| Gene ID | Gene description | log_2_(FC) | P adj | log_2_(FC) | P adj | log_2_(FC) | P adj |
| FvH4_2g12390 | lectin protein kinase family protein | **-1.023** | **<0.001** | -0.213 | 0.603 | **-0.632** | **0.035** |
| FvH4_3g03310 | S-locus lectin protein kinase family protein | **-1.471** | **0.007** | **-2.085** | **<0.001** | **-1.911** | **<0.001** |
| FvH4_3g03320 | S-locus lectin protein kinase family protein | **-3.481** | **0.003** | -0.219 | 0.115 | **-4.543** | **<0.001** |
| FvH4_3g03433 | S-domain-1 29 | -0.179 | 0.447 | **-3.058** | **0.001** | -0.257 | 0.376 |
| FvH4_3g21400 | S-locus lectin protein kinase family protein | **-0.885** | **0.026** | **-1.325** | **0.001** | **-1.066** | **0.006** |
| FvH4_4g02170 | lectin protein kinase family protein | **-9.739** | **<0.001** | **-8.712** | **<0.001** | **-2.251** | **0.028** |
| FvH4_6g12332 | S-locus lectin protein kinase family protein | **-1.337** | **0.037** | **-1.365** | **0.036** | -1.172 | 0.059 |
|  |  |  |  |  |  |  |  |
| **(b)** **Defence response** | |  |  |  |  |  |  |
|  |  | COC vs AC | | COD vs AD | | COF vs AF | |
| Gene ID | Gene description | log_2_(FC) | P adj | log_2_(FC) | P adj | log_2_(FC) | P adj |
| FvH4_3g32830 | Seven transmembrane MLO family protein | -0.132 | 0.753 | **-1.285** | **0.026** | -0.453 | 0.309 |
| FvH4_4g19710 | MLP-like protein 423 | -1.311 | 0.075 | **-2.634** | **0.002** | **-2.680** | **0.001** |
| FvH4_4g19120 | MLP-like protein 423 | -0.056 | 0.901 | **-3.703** | **<0.001** | -0.158 | 0.639 |
| FvH4_4g19700 | MLP-like protein 423 | **-2.544** | **0.002** | **-3.743** | **<0.001** | **-3.434** | **<0.001** |
| FvH4_4g18990 | MLP-like protein 423 | -0.015 | 0.988 | **-1.215** | **0.033** | -0.147 | 0.785 |
